# Electroacupuncture Regulates Endoplasmic Reticulum Stress and Ameliorates Neuronal Injury in Rats with Acute Ischemic Stroke

**DOI:** 10.1155/2021/9912325

**Published:** 2021-08-14

**Authors:** Ya-min Zhang, Hong Xu, Su-hui Chen, Hua Sun

**Affiliations:** Department of Traditional Chinese Medicine, Peking Union Medical College Hospital, Chinese Academy of Medical Sciences, Beijing 100730, China

## Abstract

Ischemic stroke is a common cause of morbidity, mortality, and disability worldwide. Electroacupuncture (EA) is an effective method for alleviating brain damage after ischemic stroke. However, the underlying mechanism has not been fully elucidated. This study aimed to determine whether endoplasmic reticulum stress (ERS) could contribute to the protective effects of EA in cerebral ischemia/reperfusion injury (CIRI) to provide a rationale for the widespread clinical use of EA. Rats were divided into the sham-operated (sham) group, the CIRI (model) group, and the EA group. Rats in the model group were subjected to middle cerebral artery occlusion (MCAO) for 2 h followed by 72 h of reperfusion. Rats with CIRI were treated daily with EA at GV20 and ST36 for a total of 3 days. The Longa scoring system and adhesive removal somatosensory test were applied to evaluate neurological deficits. Then, 2,3,5-triphenyltetrazolium chloride (TTC) staining was performed to measure the infarct volume. Immunofluorescence staining for NeuN and GFAP and terminal deoxynucleotidyl transferase- (TdT-) mediated dUTP nick-end labeling (TUNEL) staining were performed to detect apoptotic cells in brain tissue. Immunohistochemistry, quantitative real-time polymerase chain reaction (qPCR), and western blotting were used to measure the levels of ERS indicators (GRP78, CHOP/GADD153, p-eIF2*α*, and caspase 12). The results showed that EA significantly reduced the cerebral infarct volume, improved neurological function, and inhibited neuronal apoptosis. In the EA group compared with the model group, the mRNA expression levels of GRP78 were significantly increased, and the expression levels of proapoptotic proteins (CHOP/GADD153, p-eIF2*α*, and caspase 12) were significantly decreased. These results suggest that the possible mechanism by which EA protects cells against neuronal injury in CIRI may involve inhibiting endoplasmic reticulum stress.

## 1. Introduction

Stroke is considered the leading cause of disability and death, and ischemic strokes account for approximately 80% of all strokes [1]. Moreover, the incidence of ischemic stroke has been increasing, resulting in high healthcare costs and imposing a financial burden on both families and society [2, 3]. Currently, intravenous tissue-type plasminogen activator (rt-PA) is the only thrombolytic drug approved by the FDA for the treatment of ischemic stroke. Due to the narrow time window of thrombolysis treatment and the poor prognosis of this condition, the clinical application of this drug is greatly limited [4]. Therefore, there is an imminent need to find safer and more effective therapeutic approaches for stroke patients.

Early and successful restoration of blood supply is the most effective treatment for reducing the cerebral infarct volume. Paradoxically, the restoration of blood flow is frequently associated with additional brain injury, which is referred to as cerebral ischemia/reperfusion injury (CIRI) [5]. Energetic failure, calcium overload, excitotoxicity, oxidative stress, inflammation, and cell death were shown to be pathological mechanisms of CIRI [6]. Many studies have reported that apoptosis plays an important role in the pathophysiology of CIRI [7–9]. Recent studies have revealed that endoplasmic reticulum stress (ERS) is an essential signaling event for neuronal apoptosis resulting from CIRI [10]. The endoplasmic reticulum (ER) is an organelle that plays a major role in the folding, posttranslational modification, and transport and secretion of lumen and membrane proteins and maintains the Ca^2+^ balance [11]. Cerebral ischemia might cause the ER environment to become unbalanced and dysfunctional, resulting in the accumulation of unfolded/misfolded proteins in the ER lumen, which is referred to as ERS. ERS activates the apoptotic process, triggers brain damage, and impairs neurologic function [10]. According to previous studies, regulation of ERS-related apoptosis may exert a strong protective effect against CIRI [12–14].

Acupuncture is an important therapeutic approach of traditional Chinese medicine (TCM). Electroacupuncture (EA), a technique that combines traditional acupuncture with electrical stimulation, has been widely applied for the rehabilitation of stroke patients in the clinic and has achieved satisfactory effects without inducing any special side effects. Our previous studies demonstrated that the neuroprotective effects induced by EA treatment at GV20 and ST36 may involve a reduction in the inflammatory response in the brain, amelioration of damage to the blood-brain barrier (BBB), and promotion of axonal regrowth [15, 16]. Although several studies have reported the antiapoptotic capacity of EA in ischemia, this mechanism is not fully elucidated. ERS is an essential signaling event for neuronal apoptosis. Whether ERS-induced apoptosis participates in the anticerebral ischemia/reperfusion injury of EA is rarely reported. Thus, the current study intends to elaborate how EA regulates ERS against reperfusion period in cerebral ischemia.

## 2. Materials and Methods

### 2.1. Animals

Adult male SD rats (230–250 g) were purchased from Beijing Vital River Laboratory Animal Technology Co., Ltd., and housed in a controlled environment (22 ± 2°C, relative humidity of 60–70%; 12 h/12 h light/dark cycle). All rats received standard laboratory food and water ad libitum. All efforts were made to minimize animal suffering and the number of animals employed. The animal study was reviewed and approved by the Ethics Committees of Peking Union Medical College Hospital and the Chinese Academy of Medical Sciences for the Care and Use of Laboratory Animals (permit no. XHDW-2016-021).

### 2.2. CIRI Modeling

An MCAO model was established based on the methods described by Longa et al. [17]. Briefly, the rats were anesthetized by intraperitoneal injection of sodium pentobarbital (40 mg/kg). The skin was routinely disinfected, and a 2–3 cm midline incision was made in the middle of the neck. The right middle cerebral artery (MCA) was occluded with a 4-0 suture (2636-4A, Beijing Shadong Biology Company, China) with a blunted tip coated with poly-L-lysine via the internal carotid artery. The suture was advanced 18–20 mm from the carotid bifurcation to the origin of the MCA. After 2 hours, the suture was gently withdrawn under anesthesia to allow reperfusion. Sham-operated rats were subjected to the same surgical procedure without artery occlusion. The rectal temperature of the rats was maintained throughout the procedure at 37 ± 0.5°C with a temperature-regulated heating pad.

### 2.3. Grouping and Treatment

The rats were randomly divided into the sham-operated (sham) group, the CIRI model (model) group, and the EA group, with twelve rats per group. All rats except those in the sham group were subjected to transient MCAO.

The rats were placed in special cloth bags that allowed their left legs and heads to be exposed. The rats in the sham and model groups were not subjected to any treatment but were placed in cloth bags for 20 min once per day. The rats in the EA group were first treated with EA after the sutures were removed. The GV20 acupoint (on the middle top of the parietal bone) and left ST36 acupoint (5 mm below the head of fibula and 2 mm lateral to the anterior tibial tubercle) were punctured with disposable, sterile acupuncture needles (diameter = 0.30 mm, length = 25 mm, Beijing Hanyi Medical Instruments, Co., Ltd., Beijing, China) at a depth of 2-3 mm. Subsequently, the acupoints were electrically stimulated (KWD-808I, Changzhou Wujin Great Wall Medical Instrument Co., Ltd., Changzhou, China) with continuous-wave stimulation at a frequency of 2 Hz (intensity of 1 mA) for 20 min daily for a total of 3 days.

### 2.4. Measurement of the Ischemic Infarct Volume

At 72 h after reperfusion, the rats (*n* = 3 in each group) were sacrificed under deep anesthesia with sodium pentobarbital (40 mg/kg body weight; I.P.). The brains were removed and immediately frozen at –20°C for 20 min. Five 2 mm coronal slices were prepared using a rodent brain matrix. The slices were immersed in 2% TTC at 37°C for 30 min and then placed in 4% paraformaldehyde for 60 min. The slices were photographed, and the infarct size was measured with image analysis software (ImageJ, NIH) by an observer with no prior knowledge of the experiment. To account for edema and differential shrinkage resulting from tissue processing, we calculated the percentage of infarct volume as follows: ((VC − VL)/VC) × 100%, where VC represents the volume of the control hemisphere and VL is the volume of noninfarcted tissue in the lesioned hemisphere [18].

### 2.5. Behavioral Tests

All rats were subjected to behavioral tests before MCAO and 72 h after reperfusion by an investigator who was blinded to the experimental design to evaluate neurological function. The rats were assessed according to the Longa scoring system as follows [17]: 0 points, the rat had no neurological deficit; 1 point, the rat failed to extend the left forepaw fully when lifting its tail; 2 points, the rat circled to the left while walking; 3 points, the rat fell to the left while walking; 4 points, the rat could not walk spontaneously or lost consciousness. For the adhesive removal somatosensory test [19], two small pieces of adhesive-backed paper (of equal size, 113.1 mm^2^), which were used as bilateral tactile stimuli, were placed on the distal radial region on the wrist of each forelimb. Then, the rat was returned to its home cage. The time required for the rat to remove each piece of paper from its forelimbs with a cutoff time of 180 s was recorded, and each rat was subjected to five trials per day. Individual trials were separated by at least 5 min. All animals were trained three days before MCAO. Once the rats were able to remove the papers within 10 s, they were subjected to MCAO.

### 2.6. TUNEL Staining

Apoptosis was assessed by TUNEL staining. Rats (*n* = 6 per group) were anesthetized with sodium pentobarbital (40 mg/kg body weight; I.P.) and then perfused transcardially with saline (250 ml) followed by 4% paraformaldehyde (250 ml). The brains were removed, fixed in 4% paraformaldehyde at 4°C for 72 h, dehydrated, and embedded in paraffin blocks to be sliced into 3 *µ*m sections. TUNEL immunohistochemistry staining was carried out using DNA fragmentation detection with the In Situ Cell Death Detection Kit (Roche Applied Science, South San Francisco, CA, USA). Positive cells in the ischemic cortex penumbra were quantified in a blinded manner using light microscopy. The percentage of TUNEL-positive cells is presented as the ratio of TUNEL-positive cells to total cells.

### 2.7. Immunohistofluorescence Staining

The expression levels of NeuN and GFAP are correlated with pathological changes in neurons and astrocytes, respectively. Brain slices (*n* = 6 per group) were prepared and sectioned as described previously (see the TTC staining section). NeuN and GFAP immunofluorescence staining was performed as previously reported [20]. The sections were blocked in normal goat serum for 1 h at room temperature and then incubated with rabbit antibodies against GFAP (diluted 1 : 200; Abcam, UK) and NeuN (diluted 1 : 200; Abcam, UK) overnight at 4°C. Subsequently, the sections were incubated for 2 h at room temperature with fluorophore-conjugated secondary antibodies. Finally, nuclei were stained with DAPI for 10 min at room temperature. The sections were coverslipped and observed under a fluorescence microscope equipped with a digital camera. The IODs of GFAP and NeuN staining in the ischemic cortex penumbra were quantitatively analyzed.

### 2.8. Immunohistochemistry

The expression of the ERS indicators glucose-regulated protein 78 (GRP78), C/EBP homologous protein/growth arrest and DNA damage-inducible gene 153 (CHOP/GADD153), phosphorylated (p)-eukaryotic translation initiation factor 2 (eIF2*α*), and caspase 12 was measured by immunohistochemistry staining. Paraffin-embedded brain tissue sections (*n* = 6 per group) were prepared as described previously (see the TTC staining section). Briefly, sections were deparaffinized and hydrated in decreasing concentrations of alcohol and then incubated for 15 min in 1% Triton X-100 to disrupt the cell membrane. The sections were incubated with 3% H_2_O_2_ and 5% normal goat serum at room temperature for 30 min each. The brain sections were incubated overnight at 4°C with a rabbit monoclonal anti-GRP78 antibody (1 : 300, Cell Signaling Technology, USA), rabbit polyclonal anti-CHOP/GADD153 antibody (diluted 1 : 50; Sigma-Aldrich, USA), rabbit monoclonal anti-p-eIF2*α* antibody (1 : 100, Cell Signaling Technology, USA), and rabbit polyclonal anti-caspase 12 antibody (diluted 1 : 50; Abcam, UK). The rest of the protocol was performed following standard procedures. The brain sections were photographed (Leica DM4000, Germany), and the IODs of GRP78, CHOP/GADD153, p-eIF2*α*, and caspase 12 in the ischemic cortex penumbra were semiquantitatively analyzed by using image analysis software (ImageJ, NIH).

### 2.9. Western Blot Analysis

Brain tissue from the cerebral cortex in the peri-infarct region of the ipsilateral hemisphere (*n* = 3 rats per group) was dissected, immediately frozen in liquid nitrogen, and stored at −80°C for western blot analysis. The samples were homogenized in radioimmunoprecipitation assay (RIPA) lysis buffer (Beyotime Biotechnology, Jiangsu, China), and total protein was separated. The samples were separated on 12% sodium dodecyl sulfate-polyacrylamide gels, and the separated proteins were electrotransferred onto polyvinylidene fluoride membranes. After the membranes were blocked with 5% nonfat milk (BD-Becton, Dickinson and Company, San Antonio, TX, USA), they were incubated with the following primary antibodies: rabbit monoclonal anti-GRP78 antibody (1 : 1000, Cell Signaling Technology, USA), rabbit polyclonal anti-CHOP/GADD153 antibody (diluted 1 : 1000; Sigma-Aldrich, USA), rabbit monoclonal anti-p-eIF2*α* antibody (1 : 1000, Cell Signaling Technology, USA), and rabbit polyclonal anti-caspase 12 antibody (diluted 1 : 1,000; Abcam, Cambridge, UK) at 4°C overnight. The membranes were washed three times with PBST and then incubated with a species-specific horseradish peroxidase-conjugated secondary antibody (1 : 15,000, Cell Signaling Technology, USA) for 2 h. Detection was performed using an ECL kit (Millipore, USA). The density of each protein band was digitally quantified and normalized to the density of the *β*-actin band using image analysis software (Labworks 4.6, China).

### 2.10. Reverse Transcription- (RT-) Quantitative Real-Time Polymerase Chain Reaction (qPCR)

The mRNA expression levels of *GRP 78*, *CHOP/GADD153*, *p-eIF2α*, and *caspase 12* were determined by qPCR (*n* = 3 rats per group). Total RNA was extracted from the cerebral cortex in the peri-infarct region of the ipsilateral hemisphere using the RNeasy Mini Kit (Omega, USA) according to the manufacturer's protocol. The extracted RNA was then reverse-transcribed to generate cDNA. The RT reaction was performed on a Bio-Rad CFX96 Detection System (Applied Biosystems, USA) using the Plexor™ One-Step qRT-PCR System (Promega A4021, USA). The primer sequences used in this study are presented in Table 1. The fold change in relative mRNA expression was determined using the 2^−ΔΔCT^ method and using GAPDH as an endogenous reference.

### 2.11. Statistical Analysis

All quantitative data are presented as the mean ± standard deviation (SD) and were analyzed with the statistical analysis software Statistical Package for the Social Sciences (SPSS) 17.0 (IBM, Chicago, IL, USA). Data from multiple experimental groups were analyzed using one-way ANOVA followed by the least significant difference (LSD) post hoc test. In all cases, *P* < 0.05 was considered significant.

## 3. Results

### 3.1. EA Reduced the Infarct Volume and Ameliorated the Neurobehavioral Deficits in Rats with CIRI

**Figure 1:**
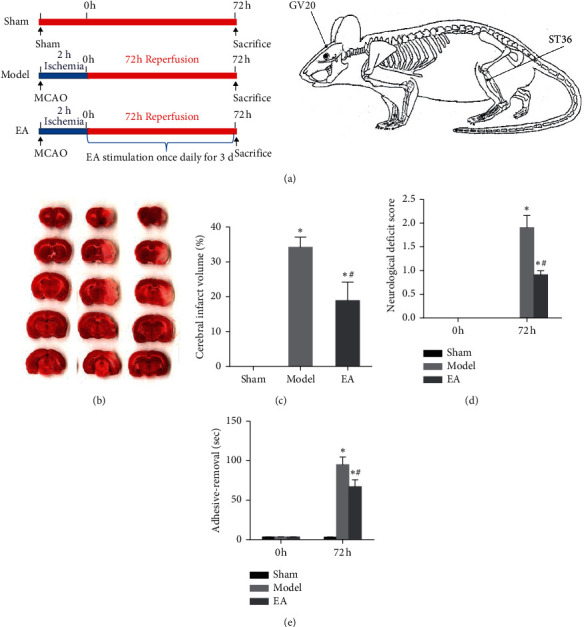
EA treatment reduced the infarct size and ameliorated the neurobehavioral deficits in rats with CIRI. (a) Schematic diagram of the experimental design. (b) Representative images of the cerebral infarct area, the size of which was assessed by TTC staining. (c) Percentage of cerebral infarct size. The data (*n* = 3) are presented as the mean ± SD. ^*∗*^*P* < 0.05 vs. the sham group. ^#^*P* < 0.05 vs. the model group. The animal neurobehavioral deficits were assessed on a 5-point scale based on the method of Zea-Longa (d) and the adhesive removal test (e) before MCAO and 72 h after reperfusion. The data (*n* = 12) are represented as the mean ± SD. ^*∗*^*P* < 0.05 vs. the sham group. ^#^*P* < 0.05 vs. the model group.

To validate the protective effect of EA treatment against CIRI, we performed middle cerebral artery occlusion (MCAO) surgery in rats as described previously [17]. The rats were randomly divided into the CIRI model (model) group and the EA treatment group, while the sham-operated (sham) group served as the control (Figure 1(a)). After 72 h of reperfusion, the infarct volume in the rats was evaluated by using 2,3,5-triphenyltetrazolium chloride (TTC) staining. The infarct lesion was stained white, while normal brain tissue was stained red (Figure 1(b)). The cerebral infarct area was significantly smaller in the EA group than in the model group (Figure 1(c)). We also examined the neurological deficits of rats. The results showed that the EA group exhibited significantly lower neurological deficit scores and better performance in the adhesive removal test than the model group at 72 h after reperfusion (Figures 1(d) and 1(e)). These results demonstrated that EA could reduce cerebral infarct volume and ameliorate neurobehavioral deficits.

### 3.2. EA Attenuated Neuronal Apoptosis and Conferred a Protective Effect against CIRI

**Figure 2:**
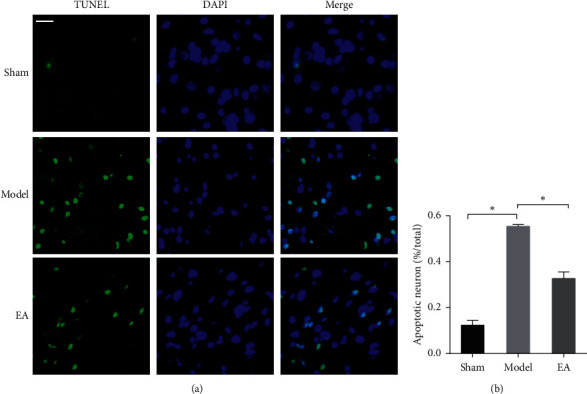
EA protects against neuronal apoptosis induced by CIRI. (a) Slices of the cortical ischemic penumbra were subjected to TUNEL staining (green). DAPI (blue) was used to counterstain the nuclei. Overlapping green and blue fluorescence indicates TUNEL-positive cells. (b) The number of TUNEL-positive cells in the different groups. Scale bar in *(a)* = 20 *µ*m (magnification 400x). The data (*n* = 6) are presented as the mean ± SD. ^*∗*^*P* < 0.05.

**Figure 3:**
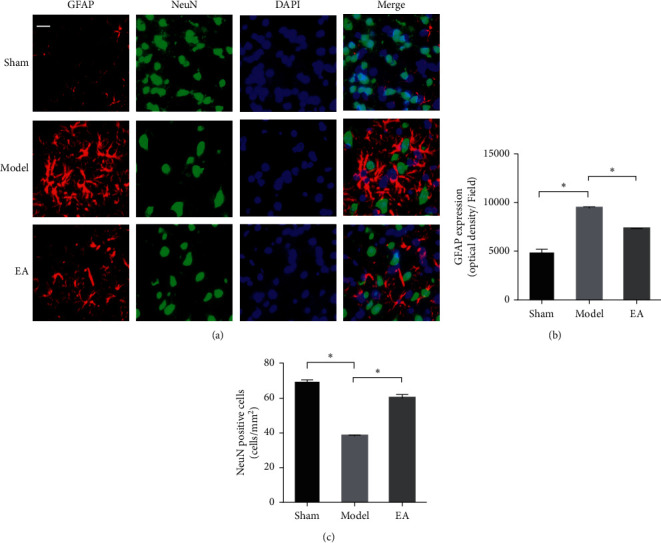
EA attenuated neuronal injury after ischemia and reperfusion. (a) GFAP-positive cells (red) and NeuN-positive cells (green) in the cortical ischemic penumbra at 72 h after reperfusion. DAPI (blue) was used to counterstain the nuclei. (b) The optical density of GFAP-positive cells in the different groups. (c) The number of NeuN-positive cells in the different groups. Scale bar in *(a)* = 20 *µ*m (magnification 400x). The data (*n* = 6) are presented as the mean ± SD. ^*∗*^*P* < 0.05.

Next, we sought to determine whether EA treatment could protect against neuronal apoptosis. Apoptotic cells were detected by terminal deoxynucleotidyl transferase- (TdT-) mediated dUTP nick-end labeling (TUNEL) staining at 72 h after reperfusion (Figure 2). TUNEL-positive cells exhibited green fluorescence and were regarded as apoptotic cells (Figure 2(a)). Very few TUNEL-positive cells were observed in the sham group. The apoptosis rate of neuronal cells was significantly lower in the EA group than in the model group (*P* < 0.05) (Figure 2(b)). NeuN-positive neurons and GFAP-positive astrocytes were also examined at 72 h after reperfusion (Figure 3(a)). GFAP expression was significantly downregulated in the EA-treated group compared to the model group (*P* < 0.05) (Figure 3(b)). However, the number of NeuN-positive cells in the EA group was significantly increased compared to that in the model group (*P* < 0.05) (Figure 3(c)). These results indicated that CIRI resulted in significant neuronal apoptosis, while EA treatment could protect against neuronal apoptosis and the activation of astrocytes.

### 3.3. EA Regulated the Expression of ERS Indicators

**Figure 4:**
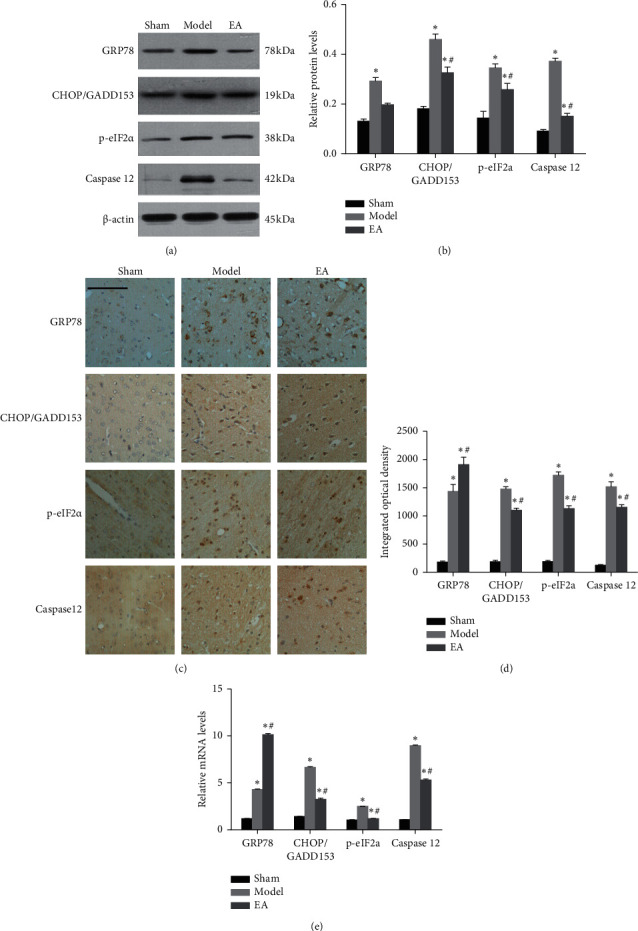
EA regulated the ERS-induced apoptotic pathway. The effects of EA on the expression of GRP78, CHOP/GADD153, p-eIF2*α*, and caspase 12 in rats were assessed by western blot analysis (a, b), immunohistochemical staining (c, d), and RT-qPCR (e) at 72 h after reperfusion. *β*-Actin served as a loading control. The mRNA levels of the genes were normalized to the GAPDH mRNA level as an endogenous reference. The data are presented as the mean ± SD. ^*∗*^*P* < 0.05 vs. the sham group. ^#^*P* < 0.05 vs. the model group. Scale bar in (c) = 100 *µ*m (magnification = 200x).

The aforementioned results indicated that EA was neuroprotective against CIRI. To evaluate the regulatory mechanisms of EA, we measured the expression levels of the ERS indicators GRP78, CHOP/GADD153, p-eIF2*α*, and caspase 12 in all groups by using western blot analysis (Figures 4(a) and 4(b)), immunohistochemical staining (Figures 4(c) and 4(d)), and qPCR (Figure 4(e)). Western blot analysis showed that the protein expression levels of CHOP/GADD153, p-eIF2*α*, and caspase 12 were significantly lower in the EA group than in the model group at 72 h after reperfusion (*P* < 0.05). The qPCR results showed that the mRNA levels of *GRP78*, *CHOP/GADD153*, *p-eIF2α*, and *caspase 12* were markedly upregulated in the model group compared with the sham group, whereas EA treatment significantly downregulated the mRNA levels of *CHOP/GADD153*, *p-eIF2α*, and *caspase 12* (*P* < 0.05). However, western blot analysis showed that the protein expression levels of GRP78 were lower in the EA group than in the model group, and the difference was not significant. However, the relative mRNA levels of *GRP78* in the EA group were significantly higher than those in the model group (*P* < 0.05). The integrated optical density results showed the same trend as the qPCR results.

## 4. Discussion

In the present study, we investigated the neuroprotective effects of EA, which attenuated brain damage and neuronal apoptosis in a rat model of CIRI induced by MCAO, as well as the molecular mechanism of its protective effect. Our studies demonstrated that EA treatment could exert significant neuroprotective effects, reduce neuronal apoptosis, and activate astrocyte-induced neuroinflammation at 72 h after reperfusion following 2 h of cerebral ischemia. Most importantly, we found that the ERS pathway could play a crucial role in the neuroprotective effect of EA against CIRI.

EA is an effective therapy for improving neurological deficits after stroke and has been widely used in the clinic for poststroke recovery [21]. The effect of EA is mainly determined by the acupoints selected. Specifically, GV20 and ST36 are two of the most effective acupoints for the treatment of ischemic stroke. GV20 belongs to the governor meridian, which is located on the top of the head. Acupuncture at GV20 is believed to nourish Qi and blood and restore consciousness. Studies have shown that acupuncture at GV20 reduces cerebral infarction following ischemia/reperfusion injury in rats [22]. ST36 is a vital acupoint of the stomach meridian of foot Yang-ming that is located at the knee joint of the lower extremities. EA at ST36 is frequently used to treat lower limb dyskinesia after stroke. Our previous studies have demonstrated that EA at GV20 and ST36 could alleviate ischemic brain damage caused by ischemic stroke through a series of mechanisms [15, 16, 23].

A complicated series of events are involved in CIRI pathology, and there is considerable evidence that apoptosis plays an essential role in this process [14]. Apoptosis is a form of programmed cell death, but it is reversible. Therefore, timely inhibition of neuronal apoptosis is an effective approach for preventing further injury induced by CIRI. In the present study, TUNEL staining was used to detect neuronal apoptosis in rats with CIRI. CIRI led to neurological dysfunction in rats and significantly increased the rate of neuronal apoptosis in these rats. We found that neurological deficits were significantly improved, the cerebral infarct volume was decreased, and neuronal cells were protected by EA treatment following CIRI. Astrocytes are specialized cells in the brain, and they play crucial roles in central nervous system homeostasis [24]. During CIRI, brain ischemia induces damage to astrocytes and stimulates the release of proinflammatory cytokines. Postischemic inflammation mediated by astrocytes was shown to be a vital contributing factor to brain injury [25, 26]. In the present study, fewer GFAP-positive cells were found in the EA group than in the model group, indicating that EA reduced the number of astrocytes after CIRI. These results suggested that EA can significantly alleviate CIRI-induced brain damage and neuroinflammation.

However, whether EA treatment attenuates CIRI by inhibiting ERS remains to be elucidated. Although mitochondrial pathways and death receptor pathways have been proposed to be the main pathways of apoptosis, ERS also contributes to apoptotic pathways in acute ischemic injury and subacute nonischemic injury caused by CIRI [10, 11, 13]. Ischemia and hypoxia in brain tissue lead to the accumulation of unfolded proteins and result in ERS. Generally, the unfolded protein response (UPR) is a self-protective response of cells that helps to reestablish ER homeostasis, but excessive activation of the UPR induces apoptosis [27]. We found that at 72 h after reperfusion, the expression of the ER apoptotic protein CHOP/GADD153, along with p-eIF2*α* and caspase 12, was significantly upregulated, suggesting that CIRI could activate ERS signaling to promote apoptosis.

ERS-induced apoptosis requires various related molecules. GRP78 is a central ER molecular chaperone. PKR-like ER kinase (PERK), inositol-requiring protein 1 (IRE1), and activating transcription factor 6 (ATF6) are three ER transmembrane receptors involved in the ERS response. Under normal conditions, GRP78 binds to PERK, IRE1, and ATF6 and inhibits their function. Under ERS conditions, GRP78 can bind to unfolded/misfolded proteins to facilitate their refolding and modification and help to restore the function of the ER. Thus, GRP78 is a sentinel marker for ERS [28]. Currently, the role of GRP78 in ERS is controversial. Previous data have indicated that downregulation of GRP78 expression may decrease ERS and reduce cell apoptosis. However, other studies have verified that activation of GRP78 decreases endoplasmic reticulum stress and neuronal death [29], and CIRI may inhibit neuronal apoptosis by upregulating GRP78 expression in the early stage of reperfusion [30]. Previous studies have found that the mRNA and protein expression levels of GRP78 induced by CIRI are different. Increased GRP78 mRNA expression was reported in the cortex and striatum at 2 h after MCAO [10], and other results confirmed that an increase in GRP78 mRNA expression was found in both the ischemic core and penumbra after MCAO, while the GRP78 protein levels decreased in the ischemic core [31]. Studies characterized in detail the cellular profiles of GRP78 expression after transient focal cerebral ischemia. GRP78 showed different patterns in the infarct and peri-infarct areas. In the infarct area, GRP78 expression was induced in almost all Iba1-positive cells, including activated microglia/macrophages, while in the peri-infarct area, GRP78 was predominantly expressed in neurons and reactive astrocytes [32]. In our study, the GRP78 mRNA expression after CIRI is consistent with the abovementioned view. However, in the WB results, the GRP78 protein level in the EA group was decreased compared with that in the model group. The difference between the ischemic penumbra and the core area was not obvious, and it is possible that the sample from the EA group contained a few ischemic cores. The results of our present study only found that EA upregulated the mRNA expression of GRP78 after 72 hours of CIRI, but we cannot conclude that the GRP78 protein level was increased by EA.

In addition, CHOP/GADD153 is an important executor in the ERS-induced apoptosis pathway [33]. The expression level of CHOP/GADD153 protein is low under unstressed conditions but increases significantly with the development of ERS, subsequently regulating intracellular Ca^2+^ metabolism and blocking B-cell lymphoma 2 (Bcl-2). Therefore, previous studies have suggested that CIRI can be alleviated by downregulating CHOP/GADD153 expression [34, 35]. In the present study, our data showed that the expression levels of CHOP/GADD153 were markedly increased in the rats with CIRI but distinctly downregulated following EA treatment. EA may inhibit the expression of CHOP/GADD153 during ERS and exert a potential neuroprotective effect.

Under ERS conditions, PERK dissociates from GRP78 and is then autophosphorylated and activated. Activated PERK phosphorylates the alpha subunit of eIF2*α*. p-eIF2*α* paradoxically increases the translation of a small subpopulation of mRNAs with short inhibitory upstream open reading frames (uORFs) in their 5′ untranslated regions, such as activating transcription factor 4 (ATF4) and CHOP/GADD153, and prolonged periods of ERS [12, 36]. According to our findings, CIRI led to increased expression levels of p-eIF2*α*, which was consistent with previous studies [12]. Of note, we found that EA intervention downregulated the expression of p-eIF2*α* and decreased apoptosis.

Caspase 12 is another major factor in the ERS-induced apoptosis pathway. Caspase 12 belongs to the conserved caspase family, which is present on the cytoplasmic surface of the ER. It has been demonstrated that the activation of caspase 12 is essential for ERS-induced apoptosis, and non-ERS-mediated apoptosis does not involve activation of caspase 12 [37–39]. When ERS occurs, Ca^2+^ release activates procaspase 12 into caspase 12 and subsequently activates caspase 9 and caspase 3, which leads to an apoptotic caspase cascade [40]. Our results were consistent with this phenomenon, and the expression levels of caspase 12 were increased in CIRI, indicating the activation of the ERS-induced apoptotic pathway. EA may have neuroprotective effects by suppressing ERS-induced apoptosis, as evidenced by a decrease in caspase 12 expression levels.

In summary, we verified that EA treatment exerts a protective effect in a rat model of CIRI and that the possible mechanism may be related to the ERS pathway and provide a theoretical basis for the clinical application of EA in ischemic stroke. However, there are still limitations in the current work. Our current study did not prove that EA can directly inhibit ERS pathways, and we only found that EA may indirectly regulate CHOP/GADD153, p-eIF2*α*, and caspase 12. Similarly, it is not clear whether EA can reduce CIRI through ERS-related inflammation, autophagy, or other signaling pathways, which is worthy of further research. Therefore, we will continue to study the possible protective mechanism of EA on CIRI, such as using key molecule inhibitors or knocking out ERS proteins to directly prove that EA can inhibit ERS pathways. These issues will be addressed in follow-up studies.

## Figures and Tables

**Table 1:** The primer sequences for qRT-PCR assays.

Gene	Primer sequences
GRP78	Forward: 5'-TGACTGGAATCCCTCCTGCTC-3' Reverse: 5'-CAGCAAACTTCTCGGCGTC-3'
CHOP/GADD153	Forward: 5'-TGCCTTTCGCCTTTGAGAC-3' Reverse: 5'-GCTTTGGGAGGTGCTTGTG-3'
p-eIF2*α*	Forward: 5'-AGGTCGCAGTTTATGTCCCTG-3' Reverse: 5'-GCTATTACCAGCACAGCAGTAGC-3'
Caspase 12	Forward: 5'-CACACTCCTGGTGTTTATGTCCC-3' Reverse: 5'-CTGTATCAGCAGTGGCTATCCC-3'
GADPH	Forward: 5'-CACAGCAAGTTCAACGGCACAG-3' Reverse: 5'-GACGCCAGTAGACTCCACGACA-3'

## Data Availability

The experimental data that support the findings of this study are available from the corresponding authors upon reasonable request.

## References

[1] Poustchi F., Amani H., Ahmadian Z. (2021). Combination therapy of killing diseases by injectable hydrogels: from concept to medical applications. *Advanced Healthcare Materials*.

[2] Wu S., Wu B., Liu M. (2019). Stroke in China: advances and challenges in epidemiology, prevention, and management. *The Lancet Neurology*.

[3] Virani S. S., Alonso A., Benjamin E. J. (2020). Heart disease and stroke statistics-2020 update: a report from the american heart association. *Circulation*.

[4] Tilling E. J., El Tawil S., Muir K. W. (2019). Do clinicians overestimate the severity of intracerebral hemorrhage?. *Stroke*.

[5] Sun M. S., Jin H., Sun X. (2018). Free radical damage in Ischemia-Reperfusion injury: an obstacle in acute ischemic stroke after revascularization therapy. *Oxidative Medicine and Cellular Longevity*.

[6] Doyle K. P., Simon R. P., Stenzel-Poore M. P. (2008). Mechanisms of ischemic brain damage. *Neuropharmacology*.

[7] Radak D., Katsiki N., Resanovic I. (2017). Apoptosis and acute brain ischemia in ischemic stroke. *Current Vascular Pharmacology*.

[8] Uzdensky A. B. (2019). Apoptosis regulation in the penumbra after ischemic stroke: expression of pro- and antiapoptotic proteins. *Apoptosis: An International Journal on Programmed Cell Death*.

[9] Gong L., Tang Y., An R., Lin M., Chen L., Du J. (2017). RTN1-C mediates cerebral ischemia/reperfusion injury via ER stress and mitochondria-associated apoptosis pathways. *Cell Death & Disease*.

[10] Nakka V. P., Gusain A., Raghubir R. (2010). Endoplasmic reticulum stress plays critical role in brain damage after cerebral ischemia/reperfusion in rats. *Neurotoxicity Research*.

[11] Zhang H.-Y., Wang Z.-g., Lu X.-H. (2015). Endoplasmic reticulum stress: relevance and therapeutics in central nervous system diseases. *Molecular Neurobiology*.

[12] Xu B., Qin Y., Li D. (2020). Inhibition of PDE4 protects neurons against oxygen-glucose deprivation-induced endoplasmic reticulum stress through activation of the Nrf-2/HO-1 pathway. *Redox Biology*.

[13] Zhu H., Fan Y., Sun H., Chen L., Man X. (2017). Curcumin inhibits endoplasmic reticulum stress induced by cerebral ischemia-reperfusion injury in rats. *Experimental and Therapeutic Medicine*.

[14] Zhao J., Li L., Fang G. (2020). Salvianolic acid a attenuates cerebral ischemia/reperfusion injury induced rat brain damage, inflammation and apoptosis by regulating miR-499a/DDK1. *American Journal of Translational Research*.

[15] Zhang Y. M., Xu H., Sun H., Chen S. H., Wang F. M. (2014). Electroacupuncture treatment improves neurological function associated with regulation of tight junction proteins in rats with cerebral ischemia reperfusion injury. *Evidence-based Complementary and Alternative Medicine: eCAM*.

[16] Chen S., Wang H., Xu H., Zhang Y., Sun H. (2020). Electroacupuncture promotes axonal regrowth by attenuating the myelin-associated inhibitors-induced RhoA/ROCK pathway in cerebral ischemia/reperfusion rats. *Brain Research*.

[17] Longa E. Z., Weinstein P. R., Carlson S., Cummins R. (1989). Reversible middle cerebral artery occlusion without craniectomy in rats. *Stroke*.

[18] Swanson R. A., Morton M. T., Tsao-Wu G., Savalos R. A., Davidson C., Sharp F. R. (1990). A semiautomated method for measuring brain infarct volume. *Journal of Cerebral Blood Flow & Metabolism*.

[19] Schallert T., Whishaw I. Q. (1984). Bilateral cutaneous stimulation of the somatosensory system in hemidecorticate rats. *Behavioral Neuroscience*.

[20] Park S. I., Park S. K., Jang K. S., Han Y. M., Kim C. H., Oh S. J. (2015). Preischemic neuroprotective effect of minocycline and sodium ozagrel on transient cerebral ischemic rat model. *Brain Research*.

[21] Chang J., Zhang H., Tan Z., Xiao J., Li S., Gao Y. (2017). Effect of electroacupuncture in patients with post-stroke motor aphasia: neurolinguistic and neuroimaging characteristics. *Wiener Klinische Wochenschrift*.

[22] Chuang C. M., Hsieh C. L., Li T. C., Lin J. G. (2007). Acupuncture stimulation at Baihui acupoint reduced cerebral infarct and increased dopamine levels in chronic cerebral hypoperfusion and ischemia-reperfusion injured sprague-dawley rats. *The American Journal of Chinese Medicine*.

[23] Wang H., Chen S., Zhang Y., Xu H., Sun H. (2019). Electroacupuncture ameliorates neuronal injury by Pink1/Parkin-mediated mitophagy clearance in cerebral ischemia-reperfusion. *Nitric Oxide*.

[24] Ding S. (2014). Dynamic reactive astrocytes after focal ischemia. *Neural Regeneration Research*.

[25] Amantea D., Micieli G., Tassorelli C. (2015). Rational modulation of the innate immune system for neuroprotection in ischemic stroke. *Frontiers in Neuroscience*.

[26] Shichita T., Ito M., Yoshimura A. (2014). Post-ischemic inflammation regulates neural damage and protection. *Frontiers in Cellular Neuroscience*.

[27] Zhang Z., Zhang L., Zhou L., Lei Y., Zhang Y., Huang C. (2019). Redox signaling and unfolded protein response coordinate cell fate decisions under ER stress. *Redox Biology*.

[28] Avila M. F., Cabezas R., Torrente D. (2013). Novel interactions of GRP78: UPR and estrogen responses in the brain. *Cell Biology International*.

[29] Louessard M., Bardou I., Lemarchand E. (2017). Activation of cell surface GRP78 decreases endoplasmic reticulum stress and neuronal death. *Cell Death & Differentiation*.

[30] Zhai M., Liu C., Li Y. (2019). Dexmedetomidine inhibits neuronal apoptosis by inducing Sigma-1 receptor signaling in cerebral ischemia-reperfusion injury. *Aging*.

[31] Ouyang Y.-B., Lu Y., Yue S. (2012). MiR-181 regulates GRP78 and influences outcome from cerebral ischemia in vitro and in vivo. *Neurobiology of Disease*.

[32] Jin X., Kim D. K., Riew T.-R., Kim H. L., Lee M.-Y. (2018). Cellular and subcellular localization of endoplasmic reticulum chaperone GRP78 following transient focal cerebral ischemia in rats. *Neurochemical Research*.

[33] Oyadomari S., Mori M. (2004). Roles of CHOP/GADD153 in endoplasmic reticulum stress. *Cell Death & Differentiation*.

[34] Timmins J. M., Ozcan L., Seimon T. A. (2009). Calcium/calmodulin-dependent protein kinase II links ER stress with Fas and mitochondrial apoptosis pathways. *Journal of Clinical Investigation*.

[35] McCullough K. D., Martindale J. L., Klotz L.-O., Aw T.-Y., Holbrook N. J. (2001). Gadd153 sensitizes cells to endoplasmic reticulum stress by down-regulating Bcl2 and perturbing the cellular redox state. *Molecular and Cellular Biology*.

[36] Cnop M., Toivonen S., Igoillo-Esteve M., Salpea P. (2017). Endoplasmic reticulum stress and eIF2*α* phosphorylation: the Achilles heel of pancreatic *β* cells. *Molecular Metabolism*.

[37] Ferri K. F., Kroemer G. (2001). Organelle-specific initiation of cell death pathways. *Nature Cell Biology*.

[38] de la Cadena S. G., Hernández-Fonseca K., Camacho-Arroyo I., Massieu L. (2014). Glucose deprivation induces reticulum stress by the PERK pathway and caspase-7- and calpain-mediated caspase-12 activation. *Apoptosis*.

[39] Wu S., Piao X., Wang N., Zhai Y. (2020). Naoluo Xintong capsule ameliorates apoptosis induced by endoplasmic reticulum stress in rats with cerebral ischemia/reperfusion injury. *Annals of Palliative Medicine*.

[40] Cai Z., Shen L., Ma H. (2015). Involvement of endoplasmic reticulum Stress-Mediated C/EBP homologous protein activation in coxsackievirus B3-Induced acute viral myocarditis. *Circulation: Heart Failure*.

